# Hierarchical Self-Distillation with Attention for Class-Imbalanced Acoustic Event Classification in Elevators

**DOI:** 10.3390/s26020589

**Published:** 2026-01-15

**Authors:** Shengying Yang, Lingyan Chou, He Li, Zhenyu Xu, Boyang Feng, Jingsheng Lei

**Affiliations:** 1 College of Computer Science and Technology, Zhejiang University of Science and Technology, Hangzhou 310023, China; syyang@zust.edu.cn (S.Y.); 222208855009@zust.edu.cn (L.C.); 222508854002@zust.edu.cn (H.L.);; 2 School of Information Science and Technology, Zhejiang Shuren University, Hangzhou 310015, China; 3 Department of Mathematics and Statistics, The University of Melbourne, Melbourne, VIC 3000, Australia

**Keywords:** acoustic anomaly detection, self-distillation, hierarchical loss optimization

## Abstract

Acoustic-based anomaly detection in elevators is crucial for predictive maintenance and operational safety, yet it faces significant challenges in real-world settings, including pervasive multi-source acoustic interference within confined spaces and severe class imbalance in collected data, which critically degrades the detection performance for minority yet critical acoustic events. To address these issues, this study proposes a novel hierarchical self-distillation framework. The method embeds auxiliary classifiers into the intermediate layers of a backbone network, creating a deep teacher–shallow student knowledge transfer paradigm optimized jointly via Kullback–Leibler divergence and feature alignment losses. A self-attentive temporal pooling layer is introduced to adaptively weigh discriminative time-frequency features, thereby mitigating temporal overlap interference, while a focal loss function is employed specifically in the teacher model to recalibrate the learning focus towards hard-to-classify minority samples. Extensive evaluations on the public UrbanSound8K dataset and a proprietary industrial elevator audio dataset demonstrate that the proposed model achieves superior performance, exceeding 90% in both accuracy and F1-score. Notably, it yields substantial improvements in recognizing rare events, validating its robustness for elevator acoustic monitoring.

## 1. Introduction

In industrial manufacturing and production systems, there is an increasingly urgent demand for the accurate monitoring and management of equipment operation status. Acoustic data, as an important source of inspection information, shows great potential for application in condition monitoring [1], predictive maintenance [2], safety and security [3], and anomaly detection [4] because of its ability to respond to the subtle changes in equipment operation in real time. As a high-frequency vertical transportation equipment used in modern buildings, the safety and reliability of elevator operation are directly related to the life safety and experience of elevator riders. Audio signals in elevator environments are of great significance for public safety monitoring, passenger behavior analysis, etc. However, the classification of acoustic events in elevator environments still suffers from the following challenges: mixed interference of multi-source signals, mechanical motion noises (e.g., friction sound of elevator car tracks), sounds of human activities, and sounds of electronic devices (e.g., beeping alarms) are highly overlapped in the time-frequency domain in the elevator’s confined space. The problem of category imbalance in data samples: some of the acoustic events that are focused on have very short durations and low frequencies, and account for less than 5% of the dataset, constituting a significant category imbalance problem.

Early research on environmental sound classification (ESC) primarily relied on feature engineering and traditional sequential models. Researchers achieved sound recognition by extracting acoustic features such as Mel-Frequency Cepstral Coefficients (MFCC), Fast Fourier Transform (FFT), and energy features in combination with Hidden Markov Models (HMM) [5], but Feature Engineering relies on manual experience and has poor generalization. The rapid advancement of deep learning led to convolutional neural networks (CNNs) and residual networks (ResNet) [6] superseding conventional approaches, thereby establishing themselves as the dominant paradigm for end-to-end classification networks. However, to achieve peak performance, convolutional neural networks often demand substantial computational resources and memory, which restricts their real-world application on devices with limited resources. To achieve a trade-off between model performance and computational efficiency, researchers have developed numerous techniques for model compression and acceleration, including pruning [7], quantization [8], Neural Architecture Search (NAS) [9] and Knowledge Distillation (KD) [10]. Among them, knowledge distillation distills the knowledge from a cumbersome teacher model into a compact student model within a teacher–student framework to achieve model compression. Zhang et al. [11] proposed self-distillation as a special distillation method, which does not need to train the complex teacher model independently; this method appends shallow classifiers to different depth intermediate layers of neural network, and then extracts knowledge from the deep classifiers to the shallow ones, so as to construct a “deep teacher–shallow student” knowledge transfer path. The knowledge is then distilled from the deep classifiers down to the shallow ones, thereby establishing a “deep teacher–shallow student” knowledge propagation pathway. Compared with traditional knowledge distillation, self-distillation reduces the training cost by transferring knowledge within the same network without the need to independently pre-train complex teacher models. Although it does not reduce parameters, self-distillation significantly enhances the classification performance through hierarchical knowledge propagation and realizes the balance between model performance improvement and training efficiency. This approach provides novel insights for high-precision deployment on edge devices.

To address the dual challenges of multi-source acoustic interference and class imbalance in elevator acoustic monitoring, this paper proposed ResNetSE-SD, a self-distillation-based acoustic classification model. We delineate the key contributions of this study as follows:

1. Shallow classifier branches are incorporated into three intermediate layers of the ResNetSE backbone, designating the deepest layer as the teacher and the shallower ones as students. The knowledge is then transferred from the teacher to the students via a combination of the KL divergence loss and the feature alignment loss.
2. To address the mixed acoustic interference in elevator environments, we introduced a self-attentive temporal pooling layer into the classifier network architecture, which enhances the ability to capture complex audio features through an adaptive weighting mechanism and effectively solves the problem of mixed interference of sound signals.
3. To mitigate data imbalance, the teacher network utilizes a hierarchical loss function derived from Focal Loss, which yields a marked gain in the model’s discriminative power for minority classes by reassigning importance to challenging samples.
4. Rigorous testing across two benchmark audio datasets—UrbanSound8K and an elevator audio dataset—demonstrates that the self-distillation approach and the two introduced modules are highly effective.

The structure of this paper is as follows. A review of prior work in audio classification is presented in Section 2. Section 3 details the architecture of the proposed ResNetSE-SD model and the formulation of its distillation loss. Section 4 presents the experimental setup, results, corresponding analysis, and ablation studies. Finally, Section 5 recapitulates the key conclusions and suggests promising avenues for subsequent research.

## 2. Related Works

### 2.1. Environmental Sound Classification

Early research on ESC relied heavily on handcrafted feature extraction combined with shallow classifiers. Typical acoustic features span several domains: time-domain (e.g., Short-Time Energy, STE), frequency-domain (e.g., Spectral Centroid), and time-frequency representations (e.g., Mel-Frequency Cepstral Coefficients [12] and wavelet transform [13]), the latter of which preserves crucial temporal information lost in standard spectrograms. These feature sets were subsequently fed into classifiers, including Support Vector Machines (SVM) [14], Hidden Markov Models (HMM) [5], and Gaussian Mixture Models (GMM). For instance, Aurino et al. [15] developed a One-Class SVM (OC-SVM) model, which leveraged features from time-frequency representations to identify anomalous sounds such as gunshots, breaking glass, and screams. Ota Yasuji et al. [16] applied timbre metrics to extend the concept of “significant differences in human hearing” to industrial environments. To bridge the gap in human auditory perception and industrial acoustic applications, timbral descriptors are combined and short-term characteristics used an SVM for binary classification. Ntalampiras et al. [17] applied a set of three generative approaches for the probabilistic detection of novel events in audio surveillance under realistic conditions, specifically the generalized GMM, generalized HMM, and GMM clustering models. Yaman et al. [18] extracted two features, Discrete Wavelet Transform (DWT) and Local Binary Pattern (LBP), from the rotor and bearing fault dataset of three-phase induction motors, employing SVM and K Nearest Neighbors (KNN) for fault detection. The above methods are effective in simple scenarios, but have limited ability to model complex noise environments and nonlinear feature relationships.

Driven by progress in deep learning, Convolutional Neural Networks (CNNs) [19], Recurrent Neural Networks (RNNs) [20], and hybrid architectures (e.g., CRNN [21]) are becoming mainstream approaches, and end-to-end acoustic classification models are gradually replacing the traditional methods. CNNs extract localized features from time-frequency graphs, such as Mel spectrograms, while RNNs can model temporal dependencies. However, the limited labeled data in industrial scenarios constrains the generalization performance of deep models. Jung et al. [22] converted the rotor faulty sounds into spectrograms via Short-Time Fourier Transform (STFT), and implemented CNNs for fault detection in industrial rotating machinery. Shevchik et al. [1] implemented the wavelet transform as an alternative to STFT-based spectrograms and used a deep CNN for real-time monitoring of additive manufacturing processes. Mu et al. [23] proposed a time-frequency attention-based CNN (TFCNN), where the time-attention mechanism and the frequency-attention mechanism focus on critical frequency bands and semantically relevant time frames in spectrograms. Jin et al. [24] proposed a Convolutional Recurrent Neural Network model (CRNN) based on the time-frequency (TF) attention mechanism and feature space (FS) attention mechanism (TFFS-CRNN). The model leverages Log-Mel spectrograms and MFCC features as inputs, which combine a time-frequency (TF) attention mechanism, a convolutional recurrent neural network (CRNN) component, a feature-space (FS) attention mechanism, and a bi-directional gated recurrent unit (BGRU) component for the recognition of overlapping sound events. Becker et al. [25] investigated LSTM networks in acoustic analysis for fault detection during 3D printing. Recent studies have further introduced self-attention mechanisms (e.g., Transformer [26]) to enhance the long-time dependency modeling capability through global context awareness. Gong et al. [27] designed an audio spectrogram transformer (AST), which was the first convolution-free, purely attention-based audio classification model, and was pretrained on the ImageNet dataset. Chen et al. [28] proposed a hierarchical Transformer architecture with windowed attention and token-semantic modules for weakly supervised sound event localization. Atito et al. [29] introduced ASiT, a self-supervised vision Transformer architecture that effectively mitigates the reliance on pre-training with image data. Vermo et al. [30] developed a framework built upon Transformer architectures for direct processing of raw audio. Despite the efficacy of Transformers in controlled environments, they encounter a dual challenge in real industrial environments: (1) requiring extensive training samples for robust feature learning and (2) susceptibility to multi-source noise, which disrupts the attention weight allocation mechanisms.

### 2.2. Knowledge Distillation

With the widening and deepening of deep learning networks, the consumption of computational resources has become increasingly prohibitive, which limits the feasibility of edge deployment. The use of lightweight neural networks with reduced parameter counts to conserve memory—which helps to expedite both training and inference processes—has emerged as a prominent research direction over the last several years. Hinton et al. first proposed the knowledge distillation framework in 2015 [10]. Knowledge distillation (KD) is a classical technique for compressing models and migrating knowledge, the essential principle of which involves distilling knowledge into a compact student network by leveraging the softened outputs from a teacher model to supervise its training. Wu et al. [31] proposed a KD technique with multi-teacher guidance, and Zhu et al. [32] proposed an online KD framework in which numerous student models are supervised by one another through ensemble techniques. Konark et al. [33] applied the KD technique in the ESC task, proposing an auto-temperature-weighted loss function that eliminates the need for manual temperature parameter tuning. Their method was validated on the ESC-10 and DCASE 2019 Task 1 (A) datasets. A literature review on KD techniques can be found in [34]. However, traditional KD requires the pre-training of independent teacher models, whose upper performance limit directly affects the student model’s performance and is expensive to train.

To overcome the limitations of traditional knowledge distillation, Zhang et al. [11] proposed the self-distillation method, which achieves self-optimization through knowledge transfer within a single network without pre-training independent teacher models. Shallow classifiers are inserted in the intermediate layers of the backbone network, and deep-to-shallow knowledge transfer is realized through the KL divergence loss and feature alignment loss. It can enhance the model representation ability and generalization performance. DKS [35] explored the possibility of using the structural knowledge of shallow layers to inversely regularize the learning of the backbone network. FRSKD [36] introduced a complex self-learning network to distill refined knowledge into a backbone. Li et al. [37] proposed a dual-teacher mechanism within the framework of self-distillation: the historical teacher utilizes predictions from historical learning phases (previous epoch) as supervisory signals and provides knowledge in the time dimension through dynamic soft labels (a weighted combination of historical predictions and ground true labels). The structural teacher employs a multi-branch network design, in which the deep backbone network guides the shallow auxiliary branches, utilizing the hierarchical features of the current network structure.

## 3. Methodology

### 3.1. Overview of ResNetSE-SD

**Figure 1:**
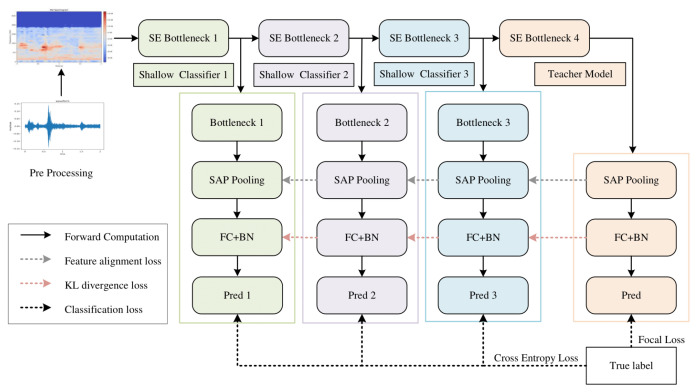
Overall architecture of the proposed ResNetSE-SD model.

The ResNetSE-SD framework proposed in this paper is shown in Figure 1, which constructs multi-level distillation paths on the ResNetSE-50 backbone network. Comparing with the original model, self-distillation inserts shallow classifier branches (Shallow Classifier1, Shallow Classifier2, and Shallow Classifier3) after each of the three intermediate layers (SE Bottleneck1, SE Bottleneck2, and SE Bottleneck3) of the ResNetSE network without modifying the structure of the backbone network. Throughout the training phase, the shallow classifiers function as student models, with the final classifier acting as the teacher. The enhanced objective function facilitates the transfer of knowledge between the teacher and student models. During testing, only the deepest classifiers are retained, and the shallow branches are automatically disabled to eliminate additional computational overhead and prevent any impact on the final classification results. Note that the backbone network can be any feature extraction network. We adopt ResNetSE-50 [38] as our backbone network, noted for its Squeeze-and-Excitation (SE) module. This module boosts the model’s feature discriminability via a channel attention mechanism that captures inter-channel dependencies within convolutional features.

The ResNetSE-SD network training process can be divided into the following modules:

Input feature preprocessing: The input audio signal undergoes Mel spectrogram transformation to generate a fixed size time-frequency representation (98 frame time steps, 80 mel bands). The time and mel bands are converted through dimensionality transformation, and the low-level acoustic features are extracted by channel expansion and initial convolution with 3 × 3 convolutional layers (output channel 32).

Teacher network: The preprocessed acoustic features undergo progressive processing through the four-stage SE Bottleneck module, which produces abstract semantic representations. These refined representations then pass through a self-attentive temporal pooling (SAP) layer and subsequently through two batch-normalized fully connected (FC) layers, generating the final predictions from the teacher network.

Student Network: Shallow Classifier1–3 are appended after SE Bottleneck1–3, which consists of a feature alignment module (Bottleneck), a SAP layer, and a feature classifier (detailed in Section 3.2), producing intermediate predictions (Pred1–3) from hierarchical features.

The proposed framework exhibits a functional symmetry through the equilibrium of its multi-objective loss function, as illustrated by the distinct paths in Figure 1. The classification loss (dark gray arrows) ensures task accuracy, the feature alignment loss (black arrows) constrains representation consistency, and the KL divergence loss (red arrows) transfers the teacher’s distributional knowledge. These components form a symbiotic and balanced supervision system.

The self-distillation loss function comprises three components. The classification loss utilizes the ground-truth labels for both networks: Focal Loss is employed to recalibrate the weighting of challenging categories in the teacher network, while the student network adopts the standard Cross Entropy Loss. The shallow classifier (student network) learns the soft labels distribution of the deeper teacher network through KL divergence loss to realize the knowledge transfer of inter-class relationships. Concurrently, an L2 alignment loss in the feature space is incorporated to enforce the similarity of representations across shallow and deep layers. The overall optimization objective, which balances the classification error, KL divergence, and feature similarity, is governed by the hyperparameters α and β.

### 3.2. Shallow Classifiers

The architecture of a shallow classifier branch comprises the following key elements: a feature alignment module, a self-attentive pooling layer, and a feature classifier. Table 1 shows the detailed descriptions of the three shallow classifiers.

Feature alignment module: spatial down-sampling and channel adjustment of intermediate features using convolutional layers to gradually down-sample the output features of each intermediate shallow classifier to the same dimension. As shown in Table 1, the feature alignment module employs convolutional layers with dynamically adjusted kernel sizes, strides, and padding to progressively down-sample the spatial dimensions of the intermediate features. Despite varying input sizes, these layers are specifically configured to produce a unified output spatial dimension of 10 × 13 (height × width) for all three stages. This ensures that the subsequent reshape operations are identical, enabling consistent feature representation for the self-attentive pooling layer.

**Figure 2:**
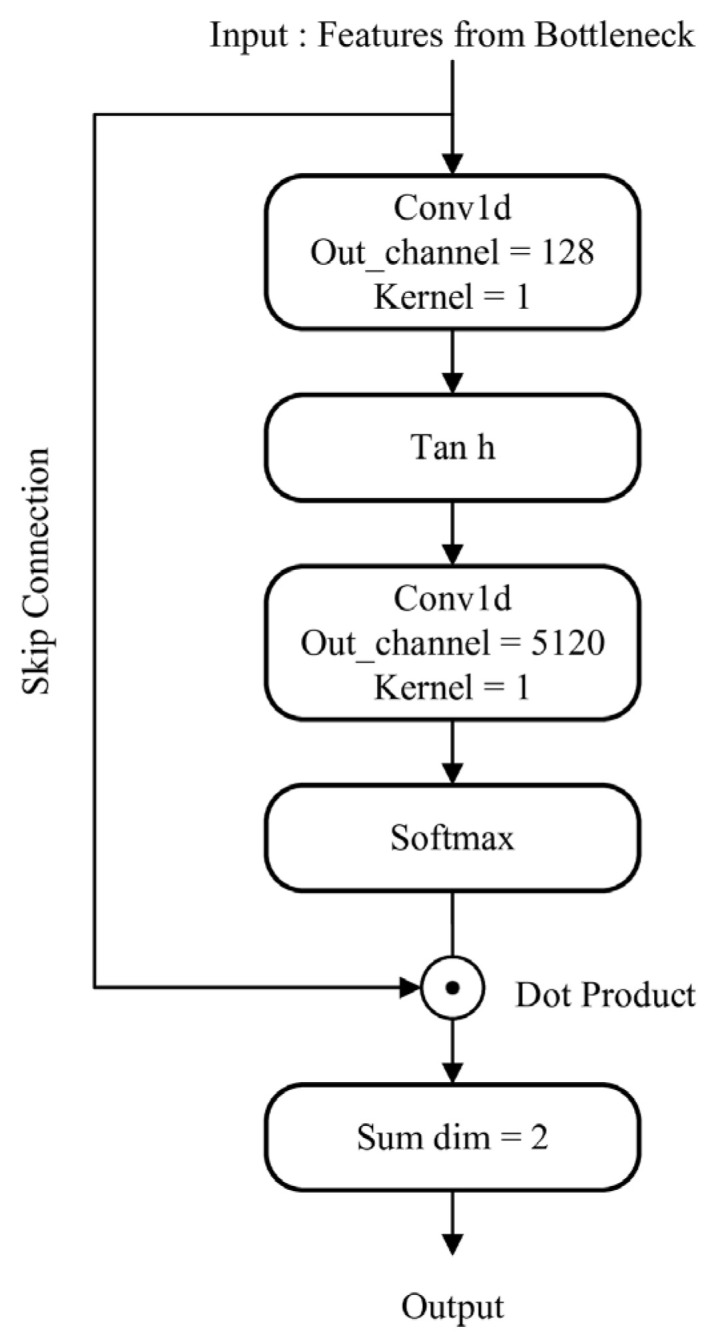
The architecture of SAP.

Self-Attentive Pooling (SAP), as illustrated in Figure 2, serves as the central module within the shallow classifiers. This method operates by leveraging an attention mechanism to perform temporal pooling. It dynamically highlights more discriminative audio features in the temporal domain by learning a self-adaptive weighting scheme. Architecturally, it employs two 1D convolutional layers—rather than linear layers—to prevent computational overhead associated with input transposition. The first convolutional layer reduces the input feature dimension to a compressed bottleneck, and its output is passed through a tanh activation to form an intermediate representation. The second convolutional layer then maps this representation back up to the input’s original dimensionality. The resulting values are normalized by a softmax function applied along the time axis, producing a set of attention weights. These weights are used in a Hadamard product with the input features, which are then summed along the temporal dimension. The final output is a fixed-size, discriminative embedding vector ready for the classification stage.

The feature classifier is built as a cascade of batch normalization (BN) and fully connected (FC) layers. This design successively employs two BN-FC stages to reduce the dimensionality of the pooled features, culminating in a final FC layer that produces the class probability distribution for the subsequent KL divergence loss calculation within the optimization objective.

### 3.3. Loss of Self-Distillation

The composite objective of our self-distillation framework integrates three key components: a classification loss, a KL divergence loss, and a feature alignment loss. Specifically, the classification term employs a tiered formulation to mitigate class imbalance, and its synergistic combination with the other two losses drives the overall optimization process.

Hierarchical classification loss: The classification loss comprises both the teacher model loss and the student model loss. To address the severe class imbalance in elevator audio data, we design a hierarchical classification loss function. The teacher network employs Focal Loss, which reconstructs the standard cross-entropy loss by introducing adjustable parameters $\alpha_{y}$ and γ. This approach reduces the loss weight for majority class samples and retains a higher loss weight for minority class samples. The calculation formulas are as follows:

(1) $$p_{te}=\frac{\exp(z_{te})}{\sum_{j=1}^{c}\exp(z_{te}^{j})}$$

(2) $$L_{teacher}=-\alpha_{y}(1-p_{te}{)}^{\gamma }\log(p_{te})$$

where c is the total number of categories, $z_{te}$ is the logit value of the teacher model output for the target category, $p_{te}$ is the predicted probability of the teacher model for the true category, $\alpha_{y}$ is the equilibrium weight for category y, and γ is the focus parameter with a default value of 2.

For each student model, the standard cross-entropy loss was used, and the total student model loss formula is as follows:

(3) $$L_{student}=\sum_{i=1}^{k}CrossEntropy(z_{st}^{\left(i\right)},y)$$

where k is the number of shallow classifiers, $z_{st}^{\left(i\right)}$ is the logit outputs from the i-th student model, and y is the ground-truth class label. Since the student model mainly captures low-level acoustic features, introducing Focal Loss at this stage may prematurely focus on hard-to-classify samples, which could amplify noise interference in feature representations. To validate this hypothesis, we conducted an ablation study comparing student models trained with Focal Loss to those trained with standard cross-entropy.

The final classification loss formula is as follows:

(4) $$L_{cls}=L_{teacher}+L_{student}$$

KL divergence loss: This loss aligns the probability distributions between student and teacher models by minimizing the Kullback–Leibler divergence (KL divergence), enabling the student model to mimic the teacher model output, which is calculated using the formula below:

(5) $$q_{te}=\frac{\exp(z_{te}/T)}{\sum \exp(z_{te}/T)}$$

(6) $$q_{i}=\frac{\exp(z_{st}^{\left(i\right)}/T)}{\sum \exp(z_{st}^{\left(i\right)}/T)}$$

(7) $$L_{kd}=\sum_{i=1}^{k}KL(q_{te},q_{i})$$

Here, T (typically set T > 1) is the temperature parameter, $z_{te}$ and $z_{st}^{\left(i\right)}$ as mentioned above are the logit outputs of the teacher and student models for the true class, $q_{te}$ and $q_{i}$ are the temperature-scaled Softmax probabilities of the teacher and student models, which are computed by the Kullback–Leibler divergence loss (KL divergence loss) and then summed up to obtain the model’s distillation loss.

Feature alignment loss: This loss enforces similarity between intermediate and final feature representations by computing the $L_{2}$ distance between the teacher’s feature maps and those of each student classifier. It injects implicit knowledge into the feature alignment modules of shallow classifiers, ensuring that student features evolve toward the teacher’s features. The formula is calculated as follows:

(8) $$L_{fea}=\sum_{i=1}^{k}L_{2}(F_{i},F_{te})$$

where $F_{i}$ is the feature map output by the student model, and $F_{te}$ is the feature map output by the teacher model, and $L_{2}$ indicates the squared $L_{2}$ norm.

Total loss function: The overall loss function combines the classification loss, KL divergence loss and feature alignment loss. The formula is calculated as follows:

(9) $$\text{Total loss}=(1-\alpha )*L_{cls}+\alpha *L_{kd}+\beta *L_{fea}$$

Here, α and β are self-distillation parameters, which balance the hierarchical classification loss, KL divergence loss, and feature alignment loss. In Section 4.5, a sensitivity analysis through ablation experiments will systematically investigate the impact of these hyperparameters on model performance.

## 4. Experiments and Results

This section presents the experimental results of our method on both the public UrbanSound8K dataset and a private industrial elevator audio dataset, benchmarked against prevalent audio networks (ResNetSE, Res2Net, CAMPPlus, ERes2Net, ERes2NetV2, TDNN, and EcapaTDNN) to substantiate its advantages; detailed ablation experiments then dissect the impact of our proposed enhancements.

### 4.1. Datasets

This study employs two audio corpora for evaluation: UrbanSound8K [39] as a public benchmark and a proprietary dataset of industrial elevator sounds.

UrbanSound8K, as a benchmark dataset for environmental sound classification, contains 8732 audio samples, each of which is no longer than 4 s in duration, and consists of 10 types of urban environment audio events, including 10 types of urban environment sound events, namely air conditioner, car horn, children_playing, dog bark, drilling, engine idling, gunshot, jackhammer, siren, and street music. Sampling rates for the audio signals vary between 16 kHz and 48 kHz. We employed a random 8:2 split for training and testing to facilitate efficient comparison across multiple baseline models. To ensure the robustness of our proposed method, we additionally conducted 10-fold cross-validation following the official dataset partitions.

We constructed an in-house elevator audio dataset of 17,203 samples extracted from surveillance system microphones in elevators within controlled, non-public university buildings, and featuring a fixed 22.05 kHz sampling rate. Given the sensitive nature of surveillance audio, all data underwent strict de-identification. Segments with human voices were processed using frequency perturbation and pitch shifting. Additionally, experts conducted sample reviews to ensure speech content remained unrecognizable. The audio signals captured two primary sound sources: elevator equipment operation noise, and sounds created by the occupants (e.g., electronic devices and conversations). The dataset contains six acoustic event classes, as detailed in Table 2, including blank, buzzer, elec_device, lift_noise, loud_talking, and regular_talking. Due to the elevator scenario’s special environment which resulted in the severe class imbalance, the blank class accounted for 59.3%, while the two key classes of abnormal acoustic events—lift_noise and loud talking—together accounted for only 6.9%. All audio samples were standardized to a 2 s duration. We employed stratified sampling to partition the data into training and test subsets with an 8:2 ratio, resulting in 13,762 and 3441 samples, respectively. This technique ensured that the category distribution was consistent across both splits, thereby guaranteeing the reliability of the evaluation.

### 4.2. Experiment Settings

All experiments were developed based on PyTorch 2.3.1 on Ubuntu 20.04.6 LTS operating system with 16 GB RAM and NVIDIA GeForce RTX 3080. Our training configuration adopted the Adam optimizer alongside a WarmupCosineSchedulerLR. The Adam optimizer is a widely adopted stochastic optimization algorithm that combines the advantages of momentum-based and adaptive learning rate methods. It maintains per-parameter learning rates updated based on estimates of the first and second moments of gradients, enabling robust convergence and efficient handling of sparse gradients. The scheduler implemented a 15-epoch linear warm-up, with learning rates bounded by a maximum of 3 × 10^−4^ and a minimum of 1 × 10^−5^. The specific optimizer parameters were configured as learning rate lr = 3 × 10^−4^ and weight_decay = 1 × 10^−4^. Consistent training settings were applied across experiments, which were as follows: 60 epochs, a batch size of 32, and a fixed random seed of 42 to ensure reproducibility. Within the hierarchical self-distillation framework, the key hyperparameters were configured as follows: the temperature parameter T for the KL divergence loss, which softens the target distributions, was set to 3; the weighting coefficients α and β in the overall loss function, balancing the main classification loss and the hierarchical knowledge distillation loss, were set to 0.8 and 0.04, respectively; and for the Focal Loss used to mitigate class imbalance, the focusing parameter γ was set to 2 and the balancing factor αᵧ was set to 1.

To assess the model’s efficacy, the metrics employed were Accuracy, Precision, Recall, and F1-score. The corresponding formulas are provided below:

(10) $$Accuracy=\frac{TP+TN}{TP+TN+FP+FN}$$

(11) $$Precision=\frac{TP}{TP+FP}$$

(12) $$Recall=\frac{TP}{TP+FN}$$

(13) $$F1=2\times \frac{Precision\times Recall}{Precision+Recall}$$

The evaluation metrics are defined as follows: True Positive (TP) refers to the count of positive instances accurately identified; True Negative (TN) represents negative instances correctly classified; False Positive (FP) indicates negative instances incorrectly assigned to the positive class; and False Negative (FN) signifies positive instances that were mistakenly predicted as negative. These four fundamental counts serve as the foundation for computing Precision, Recall, and the F1-score.

### 4.3. Feature Extraction

**Figure 3:**
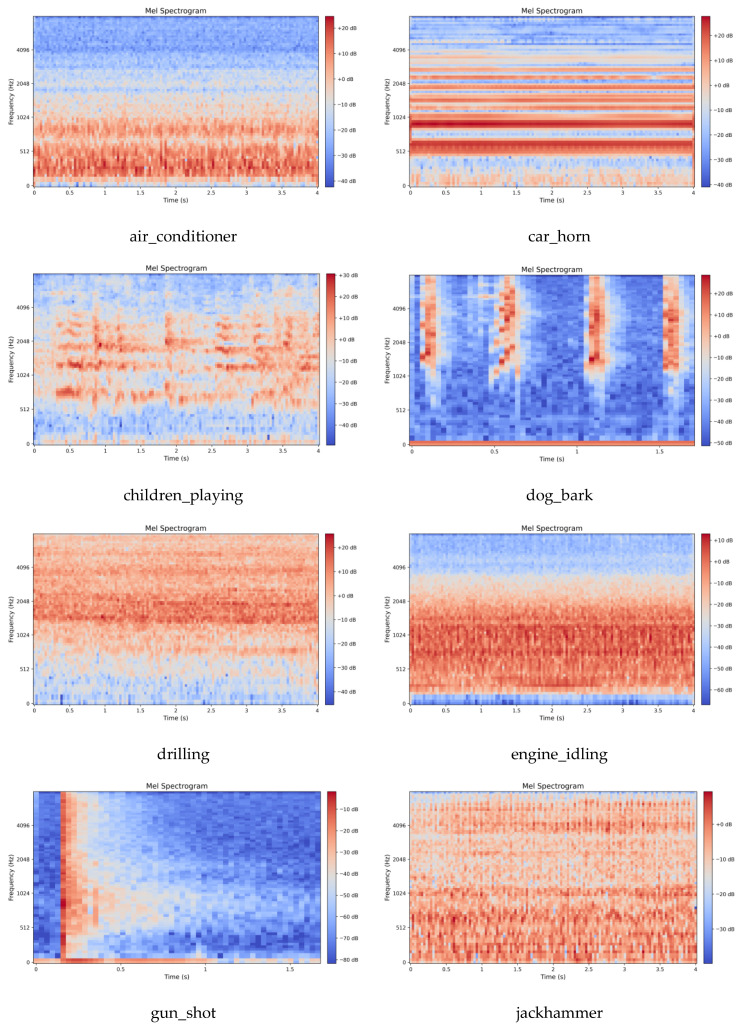

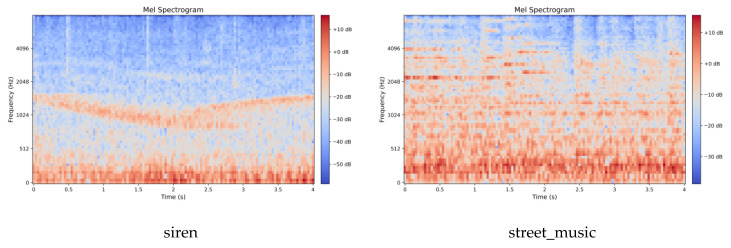
Mel Spectrogram of UrbanSound8K dataset.

**Figure 4:**
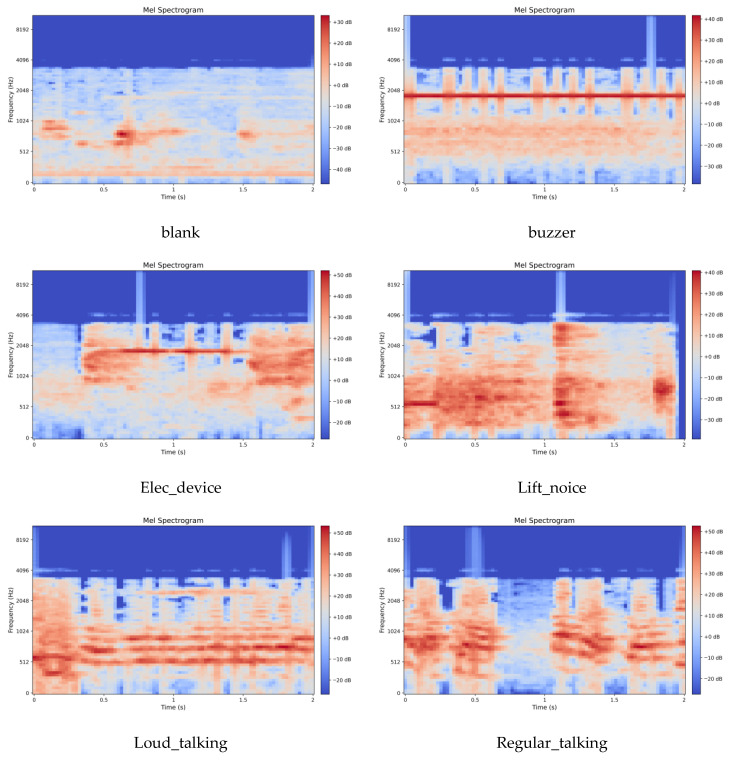
Mel Spectrogram of the elevator audio dataset.

This work employs the Mel Spectrogram for acoustic feature extraction. Mel spectrograms are time-frequency representations that map acoustic signals onto the Mel scale, a perceptually motivated frequency scale designed to approximate human auditory sensitivity. The Mel scale applies a nonlinear transformation to linear frequency (Hz), emphasizing lower frequencies where human hearing exhibits finer resolution while compressing higher frequencies. This mapping is achieved through Mel filter bank applied to the power spectrum, yielding Mel-frequency coefficients that better align with perceptual loudness and pitch discrimination. The preprocessing parameters were configured as follows: a uniform sampling rate of 16 kHz for UrbanSound8K and 22.05 kHz for the elevator dataset; an STFT Hamming window length of 2048; a frame stride of 512; and an 80-dimensional Mel filter bank. Following feature extraction, the energy values were transformed to a decibel (dB) scale via a logarithmic operation, yielding the final Mel spectrogram representation of the audio signal. The visual characteristics of the extracted Mel spectrograms for both datasets are displayed in Figure 3 and Figure 4.

### 4.4. Results of the UrbanSound8K Dataset

**Figure 5:**
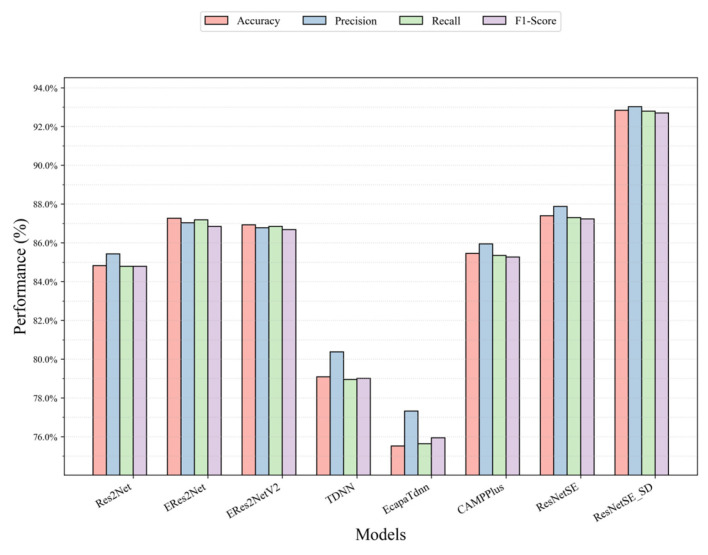
Performance comparison of all models on the UrbanSound8K dataset.

Table 2 presents a comparative analysis of the method introduced in this work against established audio classification networks (ResNetSE, Res2Net, CAMPPlus, ERes2Net, ERes2NetV2, TDNN, EcapaTDNN) on the UrbanSound8K dataset, thereby validating the efficacy of our approach. It can be observed from Table 3 that ResNetSE_SD leads significantly with an accuracy of 92.84%, which is about 5.45% higher than that of the base version of ResNetSE with an accuracy of 87.39%, and improves in the key metrics of Precision, Recall, and F1-score, with improvement values of 5.14%, 5.49%, and 5.46%, respectively, and an F1-score with the highest value of 92.69% which indicates that the model achieved the optimal balance between Precision and Recall. Recall and Precision are 92.79% and 93.02%, respectively, which are the highest values, which validates that the model effectively detects positive class samples without compromising on prediction accuracy for positive instances. In the comparison model, the accuracy rate of ERes2Net is 87.27%, which is slightly better than the accuracy rate of ERes2NetV2 (86.93%), potentially resulting in a slight performance degradation due to the simplified structure. The traditional temporal models TDNN and EcapaTdnn significantly lagged in performance, with accuracies of 79.09% and 75.52%, respectively. ResNetSE_SD significantly enhances the model’s performance capabilities by inserting shallow classifiers for intermediate layers of supervision and feature alignment, thereby verifying the effectiveness of self-distillation in audio classification tasks. Figure 5 illustrates the accuracy, Recall, Precision, and F1-scores of all models more visually.

To further validate the robustness of ResNetSE-SD, we conducted a 10-fold cross-validation experiment following the official dataset partitions. As shown in Table 4, the 10-fold cross-validation results are consistent with the random split results, with a difference of only 2.83%. The detailed per-fold results are provided in Appendix A (Table A1).

**Figure 6:**
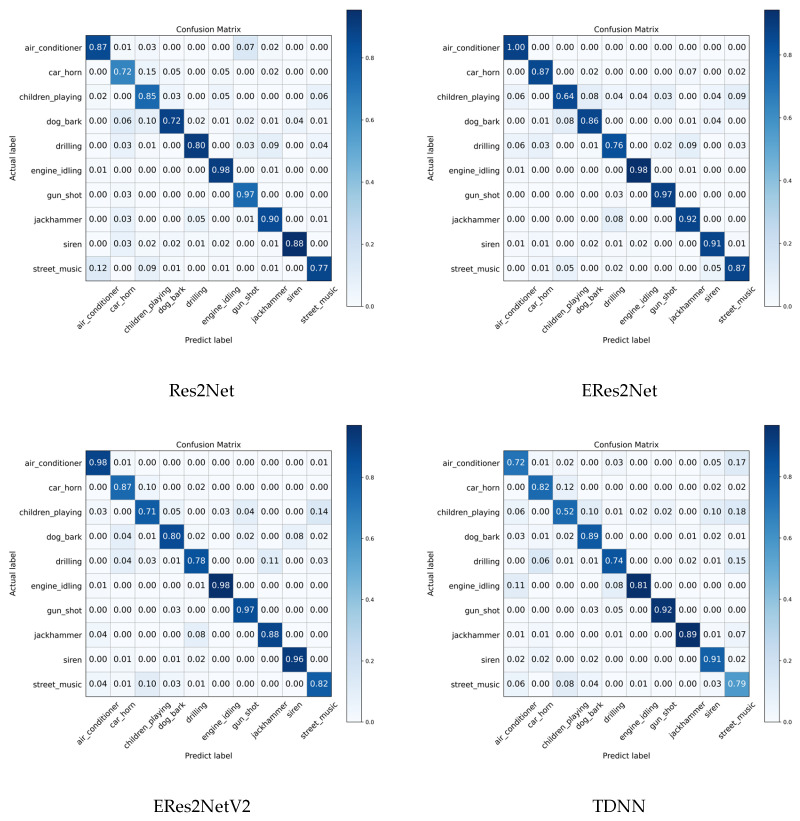

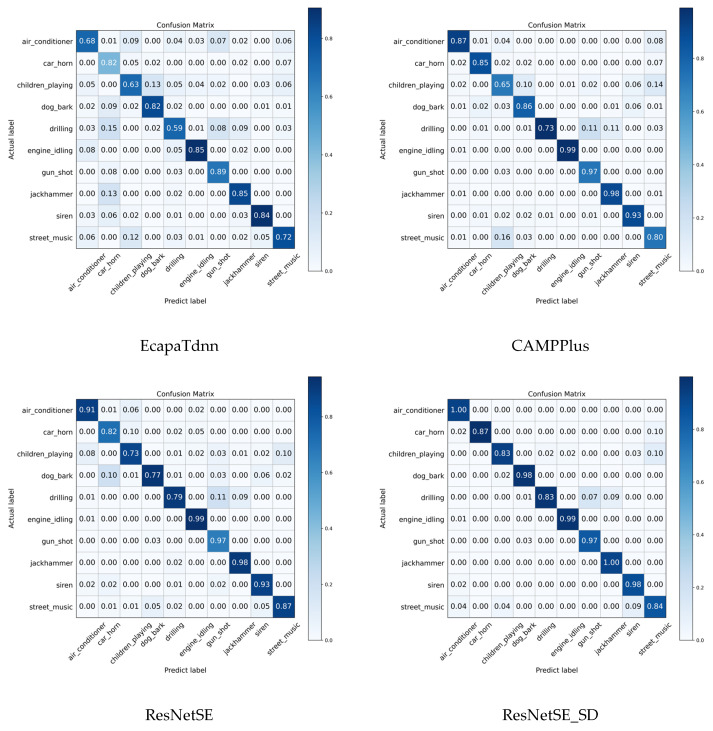
Confusion matrices of all models on UrbanSound8K dataset.

The confusion matrices for all models are presented in Figure 6. Notably, the ResNetSE_SD model achieves over 97% accuracy for categories such as air_conditioner, engine_idling, jack hammer, and gun_shot, demonstrating its proficiency in recognizing mechanical sounds. Compared to the baseline model ResNetSE, ResNetSE_SD significantly improves on most classes, with the dog_bark classification accuracy increasing from 77% to 98%, the accuracy of the children_playing classification improving from 73% to 83%, and the car_horn and siren categories showing a 5% improvement, respectively. Notably, misclassification is reduced, for example, the misclassification of the dog_bark class to car_horn is reduced from 10% to 0%, indicating that the feature alignment loss effectively suppresses sound confusion.

### 4.5. Results of the Elevator Audio Dataset

**Figure 7:**
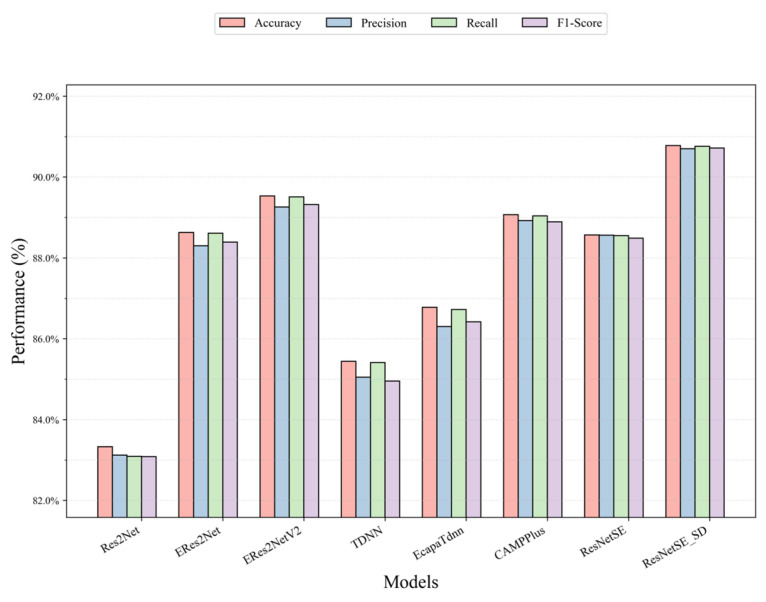
Performance comparison of all models on the elevator audio dataset.

Table 5 presents comparative results between our proposed method and commonly used audio classification networks (ResNetSE, Res2Net, CAMPPlus, ERes2Net, ERes2NetV2, TDNN, EcapaTDNN) on the elevator audio dataset to demonstrate the effectiveness of our proposed method. It can be observed from the table that ResNetSE_SD significantly leads the other models with 90.78% accuracy. Compared with the baseline model ResNetSE, ResNetSE_SD shows an improvement of 2.21% in accuracy, with simultaneous improvements in Precision (+2.14%), Recall (+2.21%), and F1-score (+2.23%), which is significantly better than its baseline ResNetSE, demonstrating the effectiveness of the distillation loss and feature alignment loss. In the comparison models, the model shows a 1.25% and 1.71% accuracy advantage over suboptimal performers ERes2NetV2 (89.53%) and CAMPPlus (89.07%), respectively, reflecting its excellent performance in the overall classification task. CAMPPlus performs the best in terms of loss values with 89.07% accuracy. TDNN and EcapaTdnn are used to process sequence data, which are the classical networks for speech recognition applications. However, they do not achieve outstanding accuracy rates, with 85.44% and 86.78%, respectively. ResNetSE_SD has the highest precision rate of 90.70% among all models, indicating its excellent performance in reducing misclassification (false positives); the recall rate is as high as 90.76%, which indicates that the model can effectively identify samples in the target category, and the risk of misclassification (false-negative) risk is significantly reduced; the F1-score leads with a combined score of 90.72%, which further validates its balance between Precision and Recall, especially applicable to scenarios with unbalanced data distribution. A comparative view of the four key metrics for various models on the elevator dataset is provided in Figure 7, offering intuitive evidence for the superiority of our framework over existing approaches in terms of Accuracy, Precision, Recall, and F1.

**Figure 8:**
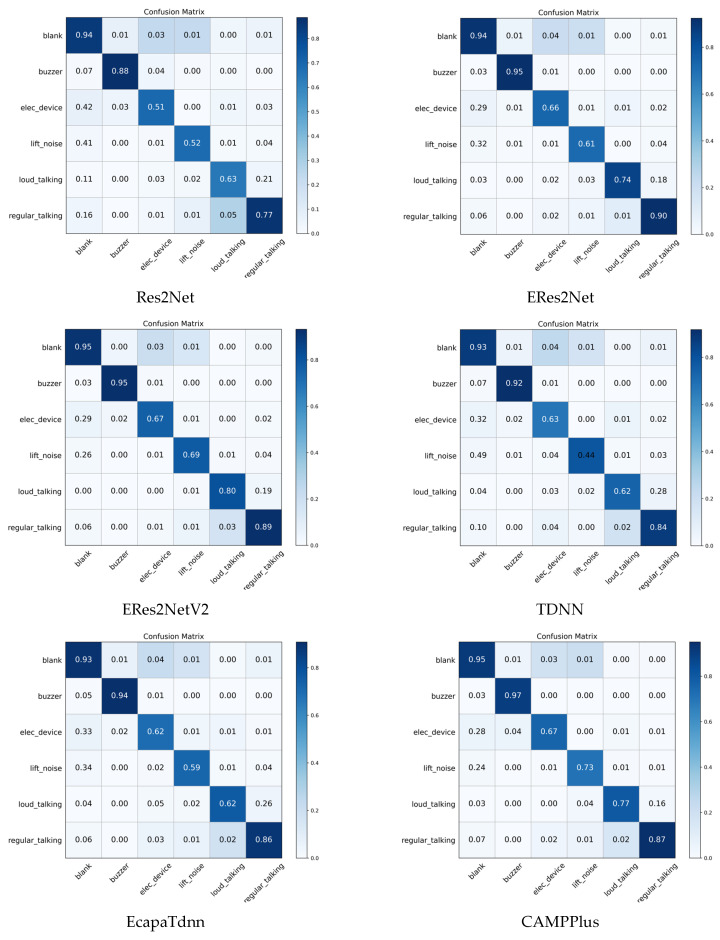

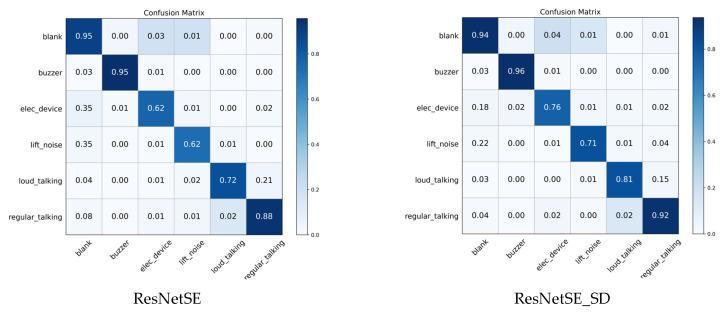
Confusion matrices for all models on the elevator audio dataset.

Figure 8 shows the confusion matrix for all evaluated models on the elevator audio dataset, revealing a clear performance difference between the majority and minority classes. For the high-frequency categories—such as blank, buzzer, and normal speech, which have percentage shares of 59.3%, 6.7%, and 16.3%, respectively—most models achieve approximately 90% recognition accuracy, demonstrating strong performance on categories with sufficient training samples. However, the key minority categories lift_noise and loud_talking, with percentage shares of only 2.1% and 4.8%, show a severe drop in accuracy: the baseline ResNetSE accuracy is only 62% and 72%, respectively, while the proposed ResNetSE_SD improves these metrics to 71% and 82%, significantly alleviating the challenge of category imbalance. The comparison model ERes2NetV2 also performs relatively well in a few categories, achieving accuracies of 69% and 80% in the classes lift_noise and loud_talking, respectively, but still not as well as ResNetSE_SD.

To comprehensively evaluate model performance under severe class imbalance, we present detailed per-class metrics and minority-class analysis. Table 6 shows Precision, Recall, and F1-score for each class across all models. Our ResNetSE-SD consistently achieves the competitive scores across all six classes. Notably, for the critical minority class lift_noise, ResNetSE-SD achieves the highest F1-score, representing a 5.8% improvement over the base ResNetSE; for the critical minority class loud_talking, ResNetSE-SD achieves the highest F1-score, outperforming all baselines.

**Figure 9:**
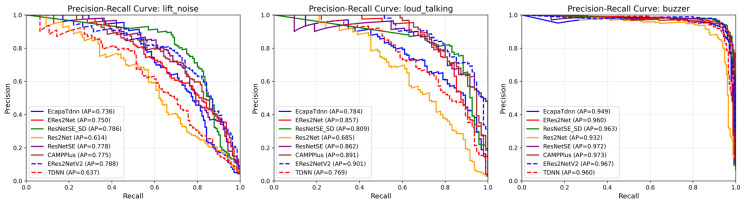
Precision-Recall curves for minority classes in elevator audio dataset.

Figure 9 presents Precision-Recall curves for the three most challenging minority classes: lift_noise, loud_talking, and buzzer. ResNetSE-SD achieves the highest Average Precision (AP) for lift_noise and a competitive AP for loud_talking.

To evaluate the computational efficiency of the proposed method, we compare the model complexity and computational requirements of ResNetSE-SD against several baseline models. Table 7 presents the number of parameters (Params) and floating-point operations (FLOPs) for each model on the elevator audio dataset. Our ResNetSE-SD model has 18.18 M parameters and requires 4.92 G FLOPs per inference.

To further quantify the overhead introduced by the auxiliary distillation branches, we measured training time and GPU memory usage. As shown in Table 8, ResNetSE-SD requires approximately 28% more training time per epoch and 12.8% more GPU memory compared to ResNetSE.

**Figure 10:**
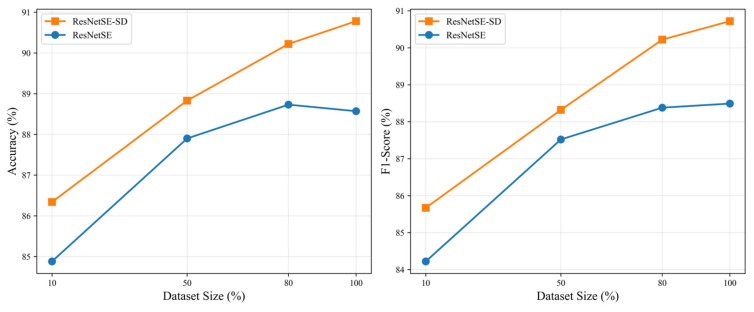
Performance trends with varying dataset sizes on elevator audio dataset.

To investigate the robustness of our proposed framework with respect to dataset size, we conducted systematic experiments on elevator audio dataset. We performed controlled experiments using stratified subsamples of varying sizes: 10%, 50%, and 80% of the original dataset. For each subsample, we maintained the original class distribution through stratified sampling and applied an 8:2 train-test split. Table 9 presents the detailed results, while Figure 10 visualizes the performance trends. Both models exhibit performance improvements as the dataset size increases, confirming the importance of sufficient training data. Notably, ResNetSE-SD consistently outperforms the baseline ResNetSE across all dataset sizes and metrics. This demonstrates the effectiveness of hierarchical self-distillation in leveraging limited data through knowledge transfer across network depths.

**Figure 11:**
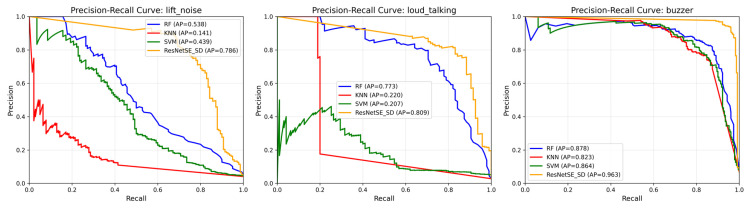
Precision-Recall curves for machine learning models comparing minority classes in elevator audio dataset.

To justify the adoption of a deep learning approach for this complex, imbalanced acoustic classification task, we compared our best model against three representative traditional machine learning methods: Support Vector Machine (SVM) with RBF kernel, Random Forest (RF), and k-Nearest Neighbors (k-NN). These models were trained on the same Mel Spectrogram features extracted from the elevator audio dataset. As shown in Table 10, traditional methods underperformed significantly compared to deep learning models. Random Forest, the best-performing traditional method, lagged behind ResNetSE-SD by approximately 13% in all metrics. Moreover, traditional methods show particularly poor performance on minority classes. Figure 11 presents Precision-Recall curves for the three most challenging minority classes, highlighting their limitation in handling class imbalance. These results validate the necessity of deep learning for complex acoustic event classification tasks like elevator monitoring.

We conducted experiments with SpecAugment [40], a state-of-the-art data augmentation technique widely used in audio classification. We implemented three key SpecAugment operations on our elevator audio dataset using the ResNetSE-SD model—Frequency Masking, which masks consecutive frequency channels with a mask ratio of 0.15; Time Masking, which masks consecutive time steps with a mask ratio of 0.2; and Time Warping, which applies temporal warping with a warp parameter of 5. As shown in Table 11, the original ResNetSE-SD model without SpecAugment achieves the best performance, outperforming all SpecAugment variants. While SpecAugment has demonstrated effectiveness on larger, more diverse datasets, our results indicate that for domain-specific industrial monitoring with limited data, our current approach achieves optimal performance without augmentation.

### 4.6. Ablation Study

To assess the contribution of core elements within the proposed framework, systematic ablation studies are performed on the audio dataset, with the impact of key hyperparameters in self-distillation also analyzed.

#### 4.6.1. Impact of Pooling Layers

Ablation analysis reveals pronounced performance differences among ResNetSE-SD models that employ various pooling strategies. The data compiled in Table 12 indicates that the self-attentive temporal pooling (SAP) configuration yields a superior accuracy of 92.84% on UrbanSound8K, exceeding alternative approaches. Similarly, for the elevator audio dataset, the SAP-equipped model achieves the best accuracy at 90.78%, albeit with a reduced margin, yet nonetheless confirms its optimal performance. These outcomes demonstrate SAP’s effectiveness in isolating salient features within complex acoustic environments, particularly those characterized by overlapping mechanical and anthropogenic sounds.

#### 4.6.2. Influence from Different Loss Function

We conducted an ablation study on the ResNetSE_SD model to verify the individual and combined effects of the proposed distillation losses, with the results presented in Table 13. The model attains 88.56% accuracy using the classification loss alone. Augmenting the objective with the KL divergence loss increases accuracy by 0.79%, underscoring the value of the knowledge encoded in the teacher’s soft labels. In contrast, adding only the feature alignment loss fails to yield significant gains, achieving an accuracy of 88.83%, which highlights its complementary nature. The holistic integration of all three losses is indispensable, culminating in a peak accuracy of 90.78% (a 1.92% gain) and affirming that the composite loss strategy is the most potent. This ablation analysis underscores the complementary roles of each loss: the KL divergence loss transfers inter-class relationships, the alignment constraint enforces feature consistency across network depths, and the classification loss anchors the learning to ground-truth labels.

**Figure 12:**
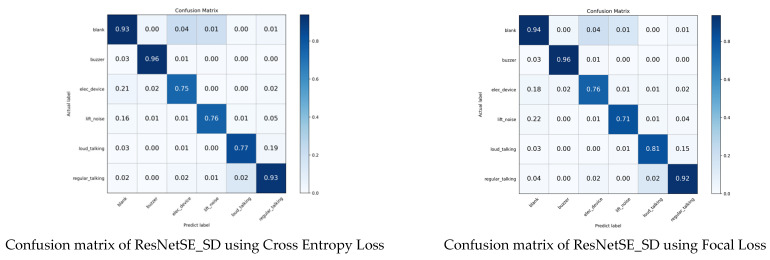
Confusion matrix of ResNetSE_SD using different loss function.

To mitigate category imbalance within the elevator audio dataset, a tiered loss strategy is introduced: the teacher network utilizes Focal Loss, whereas the student networks employ standard Cross Entropy Loss. As evidenced in Table 14, the ablation studies confirm the efficacy of this hierarchical design. The baseline model, ResNetSE_SD, achieves a classification loss accuracy of 90.34% using Cross Entropy Loss. By employing Focal Loss, the overall accuracy is improved to 90.78%. As illustrated in the confusion matrix in Figure 12, the critical enhancement lies in the minority class loud_talking (476 samples, 2.8% of total data), where accuracy rises from 77% to 81%. It proves that Focal Loss effectively mitigates the data imbalance problem through class weight adjustment.

### 4.7. Hyper-Parameters Sensitivity Study

**Figure 13:**
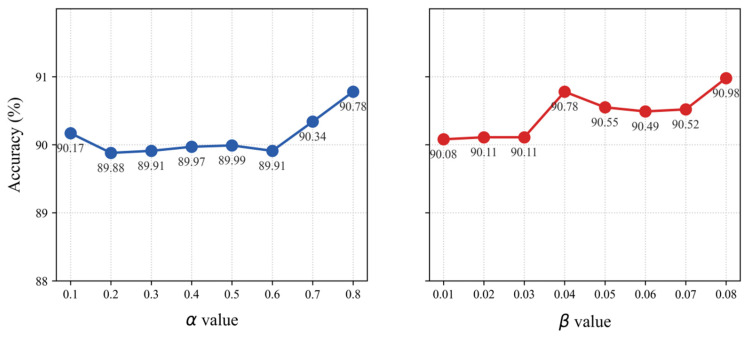
Effect of α and β on ResNetSE_SD.

The total loss function of the ResNetSE_SD model includes two new coefficients, α and β, which are used to balance its components. As documented in Figure 13, a parameter sensitivity analysis on the elevator dataset shows that accuracy spans from 89.88% to 90.78% across α values from 0.1 to 0.8. Even with the least optimal α value of 0.2, the model’s performance still exceeds that of the baseline ResNetSE. Similarly, with β varying from 0.01 to 0.08, accuracy ranges from 90.08% to 90.98%. At the lowest β value of 0.01, the self-distillation model remains more accurate than the baseline. This consistent performance across a wide range of hyperparameters validates the inherent robustness of the self-distillation framework.

## 5. Conclusions

This work has presented ResNetSE_SD, a novel model that leverages self-distillation to address the challenges of multi-source acoustic interference and class imbalance in elevator environmental sound event classification. By inserting shallow classifier branches into the ResNetSE backbone, utilizing KL divergence loss and feature alignment loss for knowledge distillation from the deepest teacher network to the shallow student network. The Self-Attentive Temporal Pooling (SAP) layers effectively solve the sound signal mixing interference problem by adaptively weighting discriminative time-frequency features. A hierarchical loss function that combines Focal Loss mitigates class imbalance by prioritizing learning for the minority class. Our model’s robust performance has been validated across both the public UrbanSound8K dataset and a complex, real-world elevator acoustic dataset.

Empirical findings show that the ResNetSE_SD model surpasses the baseline ResNetSE across all metrics and datasets, thereby validating the efficacy of the proposed self-distillation framework. Furthermore, the hierarchical loss function with Focal Loss improves the recognition of extreme minority classes compared to traditional CE Loss, and integrating this component with the KL divergence and feature alignment losses yields the highest overall accuracy.

A promising avenue for future work is to employ generative adversarial networks and diffusion models for synthesizing high-quality, multi-channel audio samples tailored to sparsely represented classes. This strategy is expected to overcome the limitations imposed by standard data augmentation in the context of unbalanced industrial elevator audio data. An in-depth study of the self-distillation mechanism would explore the knowledge transfer between different network structures [41], and try to introduce more flexible distillation paths and multi-teacher network architectures to enhance the learning ability of shallow networks for complex acoustic features. This would also advance edge deployment and real-time inference by optimizing the model for low-latency execution on embedded hardware suitable for elevator control systems. These directions aim to bridge the gap between laboratory validation and practical industrial adoption.

## Figures and Tables

**Table 1:** Structural details of three shallow classifiers.

Module	Stage1	Stage2	Stage3
Input layer	(c = 64, h = 80, w = 98)	(c = 128, h = 40, w = 49)	(c = 256, h = 20, w = 25)
Feature alignment module	Kernel Size = 7 × 7 channels = 512 Stride = 8 Padding = 3 × 3 Output: (512, h = 10, w = 13)	Kernel Size = 5 × 5 channels = 512 Stride = 4 Padding = 2 × 2 Output: (512, h = 10, w = 13)	Kernel Size = 7 × 7 channels = 512 Stride = 8 Padding = 3 × 3 Output: (512, h = 10, w = 13)
SAP layer	Reshape (1, 512 × 10, 13) Self-Attentive Pooling (5120,128)	Reshape (1, 512 × 10, 13) Self-Attentive Pooling (5120,128)	Reshape (1, 512 × 10, 13) Self-Attentive Pooling (5120,128)
Feature classifier	BatchNorm (5120) Fully Connected Layer (192) BatchNorm (192) Fully Connected Layer (6)	BatchNorm (5120) Fully Connected Layer (192) BatchNorm (192) Fully Connected Layer (6)	BatchNorm (5120) Fully Connected Layer (192) BatchNorm (192) Fully Connected Layer (6)

**Table 2:** Detailed class distribution of the elevator audio dataset.

Class	Sample Count	Percentage
blank	10,201	59.3%
buzzer	1154	6.7%
elec_device	1872	10.9%
lift_noise	700	4.1%
loud_talking	476	2.8%
regular_talking	2800	16.3%

**Table 3:** Experiment results on UrbanSound8K.

Models	Accuracy	Precision	Recall	F1-Score
Res2Net	84.82%	85.43%	84.78%	84.78%
ERes2Net	87.27%	87.03%	87.19%	86.84%
ERes2NetV2	86.93%	86.77%	86.84%	86.68%
TDNN	79.09%	80.38%	78.95%	79.01%
EcapaTdnn	75.52%	77.32%	75.63%	75.94%
CAMPPlus	85.45%	85.95%	85.35%	85.27%
ResNetSE	87.39%	87.88%	87.30%	87.23%
ResNetSE_SD	92.84%	93.02%	92.79%	92.69%

**Table 4:** ResNetSE-SD performance under different evaluation protocols on UrbanSound8K.

Evaluation Protocol	Accuracy	Precision	Recall	F1-Score
Random Split	92.84%	93.02%	92.79%	92.69%
10-Fold Cross-Validation	95.67%	96.20%	95.50%	95.50%

**Table 5:** Experiment results on elevator audio dataset.

Models	Accuracy	Precision	Recall	F1-Score
Res2Net	83.33%	83.12%	83.09%	83.08%
ERes2Net	88.63%	88.30%	88.61%	88.39%
ERes2NetV2	89.53%	89.26%	89.51%	89.32%
TDNN	85.44%	85.05%	85.41%	84.95%
EcapaTdnn	86.78%	86.30%	86.72%	86.42%
CAMPPlus	89.07%	88.92%	89.04%	88.89%
ResNetSE	88.57%	88.56%	88.55%	88.49%
ResNetSE_SD	90.78%	90.70%	90.76%	90.72%

**Table 6:** Per-class performance metrics on elevator audio dataset.

Models	Metric	Blank	Buzzer	Elec_Device	Lift_Noise	Loud_Talking	Regular_Talking
Res2Net	Precision	85.14%	88.65%	69.31%	68.22%	63.83%	87.22%
	Recall	93.53%	87.88%	51.20%	52.14%	63.16%	76.79%
	F1-Score	89.14%	88.26%	58.90%	59.11%	63.49%	81.67%
ERes2Net	Precision	90.78%	92.44%	73.45%	71.67%	85.37%	91.96%
	Recall	94.12%	95.24%	66.40%	61.43%	73.68%	89.82%
	F1-Score	92.42%	93.82%	69.75%	66.15%	79.10%	90.88%
ERes2NetV2	Precision	91.41%	93.22%	75.90%	77.60%	80.85%	92.91%
	Recall	94.90%	95.24%	67.20%	69.29%	80.00%	88.93%
	F1-Score	93.12%	94.22%	71.29%	73.21%	80.42%	90.88%
TDNN	Precision	87.77%	91.77%	67.24%	78.21%	73.75%	87.48%
	Recall	93.19%	91.77%	62.93%	43.57%	62.11%	83.57%
	F1-Score	90.39%	91.77%	65.01%	55.96%	67.43%	85.48%
EcapaTdnn	Precision	89.56%	90.79%	69.64%	72.17%	75.64%	88.64%
	Recall	93.38%	93.94%	62.40%	59.29%	62.11%	86.43%
	F1-Score	91.43%	92.34%	65.82%	65.10%	68.21%	87.52%
CAMPPlus	Precision	91.12%	89.56%	76.00%	70.83%	80.22%	95.10%
	Recall	94.61%	96.54%	66.67%	72.86%	76.84%	86.61%
	F1-Score	92.83%	92.92%	71.02%	71.83%	78.49%	90.65%
ResNetSE	Precision	91.28%	89.52%	70.98%	75.41%	82.35%	94.16%
	Recall	93.33%	96.10%	73.07%	65.71%	73.68%	86.43%
	F1-Score	92.29%	92.69%	72.01%	70.23%	77.78%	90.13%
ResNetSE_SD	Precision	93.67%	93.67%	75.80%	81.30%	81.91%	92.27%
	Recall	94.36%	96.10%	76.00%	71.43%	81.05%	91.61%
	F1-Score	94.02%	94.87%	75.90%	76.05%	81.48%	91.94%

**Table 7:** Model complexity comparison on elevator audio dataset.

Models	Params (M)	FLOPs (G)
Res2Net	4.16	0.11
ERes2Net	6.61	3.36
ERes2NetV2	5.46	2.10
TDNN	2.54	0.42
EcapaTdnn	5.71	0.94
CAMPPlus	7.18	1.11
ResNetSE	6.82	3.66
ResNetSE_SD	18.18	4.92

**Table 8:** Computational overhead of auxiliary branches.

Meric	ResNetSE	ResNetSE-SD	Overhead
Training time/epoch	25 s	32 s	+28%
GPU memory (peak)	2630 MB	2968 MB	+12.8%

**Table 9:** Performance comparison with varying dataset sizes on elevator audio dataset.

Models	Metric	10%	50%	80%	100%
ResnetSE	Accuracy	84.88%	87.90%	88.73%	88.57%
	Precision	84.53%	87.47%	88.35%	88.57%
	Recall	84.88%	87.85%	88.70%	88.57%
	F1-Score	84.22%	87.52%	88.38%	88.57%
ResNetSE_SD	Accuracy	86.34%	88.83%	90.22%	90.78%
	Precision	85.91%	88.37%	90.26%	90.78%
	Recall	86.33%	88.78%	90.44%	90.78%
	F1-Score	85.67%	88.32%	90.22%	90.78%

**Table 10:** Performance comparison with traditional machine learning methods on the elevator audio dataset.

Models	Accuracy	Precision	Recall	F1-Score
SVM (RBF)	46.47%	67.09%	46.47%	51.13%
Random Forest	79.72%	79.75%	79.72%	78.30%
k-NN (k = 5)	63.62%	59.46%	63.62%	54.71%
ResNetSE_SD	90.78%	90.70%	90.76%	90.72%

**Table 11:** Performance comparison of ResNetSE-SD with different SpecAugment configurations on elevator audio dataset.

SpecAugment Configuration	Accuracy	Precision	Recall	F1-Score
Frequency Masking	89.79%	89.48%	89.77%	89.45%
Time Masking	89.82%	89.55%	89.79%	89.44%
Time Warping	89.76%	89.69%	89.74%	89.65%
No Augmentation	90.78%	90.70%	90.76%	90.72%

**Table 12:** Performance of ResNetSE_SD model with different pooling layers.

Pooling Layer	UrbanSound8K Accuracy (%)	Elevator Audio Dataset Accuracy (%)
ASP	89.32%	90.28%
TAP	87.84%	89.73%
TSP	89.20%	90.28%
SAP (our model)	92.84%	90.78%

**Table 13:** Classification accuracy of ResNetSE_SD using different loss functions.

Classification Loss	KL Divergence Loss	Feature Alignment Loss	Accuracy of ResNetSE_SD
✔			88.86%
✔	✔		89.65%
✔		✔	88.83%
✔	✔	✔	90.78%

**Table 14:** Accuracy of ResNetSE_SD using different losses on elevator audio dataset.

Loss Function	Accuracy
CrossEntropy Loss	90.34%
Focal Loss	90.78%

## Data Availability

The UrbanSound8K dataset [39] is publicly accessible at https://audeering.github.io/datasets/datasets/urbansound8k.html (Access on 18 November 2025). The Elevator Audio Dataset is available upon reasonable request to the corresponding author, subject to institutional review and data-sharing agreements. Source codes are provided in Supplementary Materials.

## Supplementary Materials

The following supporting information can be downloaded at: https://www.mdpi.com/article/10.3390/s26020589/s1, the source code for implementing and training the ResNetSE-SD model and baseline models.

## Appendix A

**Table A1:** Detailed 10-fold cross-validation results of ResNetSE-SD on UrbanSound8K.

Fold	Accuracy	Precision	Recall	F1-Score
1	95.45%	96.34%	95.45%	95.27%
2	94.79%	94.99%	94.38%	94.44%
3	90.63%	91.13%	90.32%	90.21%
4	97.12%	97.40%	96.97%	96.95%
5	96.88%	97.38%	96.81%	96.87%
6	95.45%	97.10%	95.18%	95.58%
7	97.73%	98.02%	97.62%	97.67%
8	96.59%	96.84%	96.30%	96.26%
9	95.45%	95.51%	95.12%	95.02%
10	96.59%	96.93%	96.43%	96.31%

**Table A2:** Res2Net Numerical Confusion Matrix on elevator audio dataset.

	Blank	Buzzer	Elec_Device	Lift_Noise	Loud_Talking	Regular_Talking
blank	1908	16	64	24	3	25
buzzer	16	203	10	1	1	0
elec_device	158	10	192	0	3	12
lift_noise	58	0	2	73	1	6
loud_talking	10	0	3	2	60	20
regular_talking	91	0	6	7	26	430

**Table A3:** ERes2Net Numerical Confusion Matrix on elevator audio dataset.

	Blank	Buzzer	Elec_Device	Lift_Noise	Loud_Talking	Regular_Talking
blank	1920	12	72	24	1	11
buzzer	7	220	3	0	0	1
elec_device	107	5	249	2	3	9
lift_noise	45	1	2	86	0	6
loud_talking	3	0	2	3	70	17
regular_talking	33	0	11	5	8	503

**Table A4:** ERes2NetV2 Numerical Confusion Matrix on elevator audio dataset.

	Blank	Buzzer	Elec_Device	Lift_Noise	Loud_Talking	Regular_Talking
blank	1936	9	69	18	0	8
buzzer	7	220	3	0	0	1
elec_device	107	7	252	2	1	6
lift_noise	36	0	1	97	1	5
loud_talking	0	0	0	1	76	18
regular_talking	32	0	7	7	16	498

**Table A5:** TDNN Numerical Confusion Matrix on elevator audio dataset.

	Blank	Buzzer	Elec_Device	Lift_Noise	Loud_Talking	Regular_Talking
blank	1901	12	85	13	2	27
buzzer	17	212	2	0	0	0
elec_device	120	6	236	0	4	9
lift_noise	68	1	5	61	1	4
loud_talking	4	0	3	2	59	27
regular_talking	56	0	20	2	14	468

**Table A6:** EcapaTdnn Numerical Confusion Matrix on elevator audio dataset.

	Blank	Buzzer	Elec_Device	Lift_Noise	Loud_Talking	Regular_Talking
blank	1905	16	73	19	1	26
buzzer	12	217	2	0	0	0
elec_device	123	6	234	4	3	5
lift_noise	47	0	3	83	1	6
loud_talking	4	0	5	2	59	25
regular_talking	36	0	19	7	14	484

**Table A7:** CAMPPlus Numerical Confusion Matrix on elevator audio dataset.

	Blank	Buzzer	Elec_Device	Lift_Noise	Loud_Talking	Regular_Talking
blank	1930	12	62	30	1	5
buzzer	7	223	1	0	0	0
elec_device	106	14	250	0	2	3
lift_noise	33	0	2	102	1	2
loud_talking	3	0	0	4	73	15
regular_talking	39	0	14	8	14	485

**Table A8:** ResNetSE Numerical Confusion Matrix on elevator audio dataset.

	Blank	Buzzer	Elec_Device	Lift_Noise	Loud_Talking	Regular_Talking
blank	1904	19	88	15	1	13
buzzer	6	222	3	0	0	0
elec_device	88	7	274	4	1	1
lift_noise	43	0	4	92	1	0
loud_talking	5	0	2	2	70	16
regular_talking	40	0	15	9	12	484

**Table A9:** ResNetSE_SD Numerical Confusion Matrix on elevator audio dataset.

	Blank	Buzzer	Elec_Device	Lift_Noise	Loud_Talking	Regular_Talking
blank	1925	8	75	18	0	14
buzzer	6	222	3	0	0	0
elec_device	69	7	285	3	2	9
lift_noise	31	0	1	100	2	6
loud_talking	3	0	0	1	77	14
regular_talking	21	0	12	1	13	513

## References

[1] Shevchik S.A., Masinelli G., Kenel C., Leinenbach C., Wasmer K. (2019). Deep learning for in situ and real-time quality monitoring in additive manufacturing using acoustic emission. IEEE Trans. Ind. Inform..

[2] Keleko A.T., Kamsu-Foguem B., Ngouna R.H., Tongne A. (2022). Artificial intelligence and real-time predictive maintenance in industry 4.0: A bibliometric analysis. AI Ethics.

[3] Glentis G.O., Angelopoulos K., Nikolaidis S., Kousiopoulos G.P., Porlidas D., Gkountis D. Study of the acoustic noise in pipelines carrying oil products in a refinery establishment. Proceedings of the 23rd Pan-Hellenic Conference on Informatics.

[4] Lei J., Sun W., Fang Y., Ye N., Yang S., Wu J. (2024). A Model for Detecting Abnormal Elevator Passenger Behavior Based on Video Classification. Electronics.

[5] Rabiner L.R. (2002). A tutorial on hidden markov models and selected applications in speech recognition. Proc. IEEE.

[6] He K., Zhang X., Ren S., Sun J. Deep residual learning for image recognition. Proceedings of the IEEE Conference on Computer Vision and Pattern Recognition.

[7] Han S., Mao H., Dally W.J. (2015). Deep compression: Compressing deep neural networks with pruning, trained quantization and huffman coding. arXiv.

[8] Zhou Y., Moosavi-Dezfooli S.-M., Cheung N.-M., Frossard P. Adaptive quantization for deep neural network. Proceedings of the AAAI Conference on Artificial Intelligence.

[9] Elsken T., Metzen J.H., Hutter F. (2019). Neural architecture search: A survey. J. Mach. Learn. Res..

[10] Hinton G., Vinyals O., Dean J. (2015). Distilling the knowledge in a neural network. arXiv.

[11] Zhang L., Bao C., Ma K. (2021). Self-distillation: Towards efficient and compact neural networks. IEEE Trans. Pattern Anal. Mach. Intell..

[12] Abdul Z.K., Al-Talabani A.K. (2022). Mel frequency cepstral coefficient and its applications: A review. IEEE Access.

[13] Pathak R.S. (2009). The Wavelet Transform.

[14] Hearst M.A., Dumais S.T., Osuna E., Platt J., Scholkopf B. (1998). Support vector machines. IEEE Intell. Syst. Appl..

[15] Aurino F., Folla M., Gargiulo F., Moscato V., Picariello A., Sansone C. One-class svm based approach for detecting anomalous audio events. Proceedings of the 2014 International Conference on Intelligent Networking and Collaborative Systems.

[16] Ota Y., Unoki M. (2023). Anomalous sound detection for industrial machines using acoustical features related to timbral metrics. IEEE Access.

[17] Ntalampiras S., Potamitis I., Fakotakis N. (2011). Probabilistic novelty detection for acoustic surveillance under real-world conditions. IEEE Trans. Multimed..

[18] Yaman O. (2021). An automated faults classification method based on binary pattern and neighborhood component analysis using induction motor. Measurement.

[19] LeCun Y., Boser B., Denker J.S., Henderson D., Howard R.E., Hubbard W., Jackel L.D. (1989). Backpropagation applied to handwritten zip code recognition. Neural Comput..

[20] Elman J.L. (1990). Finding structure in time. Cogn. Sci..

[21] Shi B., Bai X., Yao C. (2016). An end-to-end trainable neural network for image-based sequence recognition and its application to scene text recognition. IEEE Trans. Pattern Anal. Mach. Intell..

[22] Jung H., Choi S., Lee B. (2023). Rotor fault diagnosis method using cnn-based transfer learning with 2d sound spectrogram analysis. Electronics.

[23] Mu W., Yin B., Huang X., Xu J., Du Z. (2021). Environmental sound classification using temporal-frequency attention based convolutional neural network. Sci. Rep..

[24] Jin Y., Wang M., Luo L., Zhao D., Liu Z. (2022). Polyphonic sound event detection using temporal-frequency attention and feature space attention. Sensors.

[25] Becker P., Roth C., Roennau A., Dillmann R. Acoustic anomaly detection in additive manufacturing with long short-term memory neural networks. Proceedings of the 2020 IEEE 7th International Conference on Industrial Engineering and Applications (ICIEA).

[26] Vaswani A., Shazeer N., Parmar N., Uszkoreit J., Jones L., Gomez A.N., Kaiser L., Polosukhin I. (2017). Attention is all you need. Adv. Neural Inf. Process. Syst..

[27] Gong Y., Chung Y.-A., Glass J. (2021). Ast: Audio spectrogram transformer. arXiv.

[28] Chen K., Du X., Zhu B., Ma Z., Berg-Kirkpatrick T., Dubnov S. Hts-at: A hierarchical token-semantic audio transformer for sound classification and detection. Proceedings of the ICASSP 2022—2022 IEEE International Conference on Acoustics, Speech and Signal Processing (ICASSP).

[29] Atito S., Awais M., Wang W., Plumbley M.D., Kittler J. (2024). Asit: Local-global audio spectrogram vision transformer for event classification. IEEE/ACM Trans. Audio Speech Lang. Process..

[30] Verma P., Berger J. (2021). Audio transformers: Transformer architectures for large scale audio understanding. adieu convolutions. arXiv.

[31] Wu M.-C., Chiu C.-T., Wu K.-H. Multi-teacher knowledge distillation for compressed video action recognition on deep neural networks. Proceedings of the ICASSP 2019—2019 IEEE International Conference on Acoustics, Speech and Signal Processing (ICASSP).

[32] Zhu X., Gong S. (2018). Knowledge distillation by on-the-fly native ensemble. Adv. Neural Inf. Process. Syst..

[33] Tripathi A.M., Paul K. (2022). Data augmentation guided knowledge distillation for environmental sound classification. Neurocomputing.

[34] Wang L., Yoon K.-J. (2021). Knowledge distillation and student-teacher learning for visual intelligence: A review and new outlooks. IEEE Trans. Pattern Anal. Mach. Intell..

[35] Sun D., Yao A., Zhou A., Zhao H. Deeply-supervised knowledge synergy. Proceedings of the IEEE/CVF Conference on Computer Vision and Pattern Recognition.

[36] Ji M., Shin S., Hwang S., Park G., Moon I.-C. Refine myself by teaching myself: Feature refinement via self-knowledge distillation. Proceedings of the IEEE/CVF Conference on Computer Vision and Pattern Recognition.

[37] Li Z., Li X., Yang L., Song R., Yang J., Pan Z. (2024). Dual teachers for self-knowledge distillation. Pattern Recognit..

[38] Hu J., Shen L., Sun G. Squeeze-and-excitation networks. Proceedings of the IEEE Conference on Computer Vision and Pattern Recognition.

[39] Salamon J., Jacoby C., Bello J.P. A dataset and taxonomy for urban sound research. Proceedings of the 22nd ACM International Conference on Multimedia.

[40] Park D.S., Chan W., Zhang Y., Chiu C.C., Zoph B., Cubuk E.D., Le Q.V. (2019). SpecAugment: A Simple Data Augmentation Method for Automatic Speech Recognition. arXiv.

[41] Hu H., Yang S., Zhang Y., Wu J., He L., Lei J. (2025). MetaRes-DMT-AS: A Meta-Learning Approach for Few-Shot Fault Diagnosis in Elevator Systems. Sensors.

